# Mental health symptoms and inflammatory markers among HIV infected patients in Tanzania

**DOI:** 10.1186/s12889-021-11064-5

**Published:** 2021-06-10

**Authors:** Peter Memiah, Lillian Nkinda, Mtebe Majigo, Felix Humwa, Zelalem T. Haile, Kennedy Muthoka, Aisha Zuheri, Anne Kamau, Lucy Ochola, Gabriel Buluku

**Affiliations:** 1 Division of Epidemiology and Prevention: Institute of Human Virology, University of Maryland School of Medicine, 725 West Lombard Street, Room N459, Baltimore, MD 21201 USA; 2 Department of Medicine, University of Maryland Medical Centre Midtown Campus, Baltimore, MD USA; 3 Department of Microbiology and Immunology, Muhimbili University of Health and Allied Sciences, Dar es Salaam, Tanzania; 4 Global Program for Research Teaching, University of California San Francisco, Nairobi, Kenya; 5 Department of Social Medicine, Ohio University Heritage College of Osteopathic Medicine, Dublin, OH USA; 6 Palladium Group, Nairobi, Kenya; 7 Infectious Disease Centre, Dar es Salaam, Tanzania; 8 University of Nairobi, Institute for Development Studies, Nairobi, Kenya; 9 Institute of Primate Research, Nairobi, Kenya

**Keywords:** Mental health, Inflammatory markers, People living with HIV, Antiretroviral, Dar Es Salaam

## Abstract

**Background:**

HIV and mental disorders are predicted to be the leading causes of illness worldwide by the year 2030. HIV-infected patients are at increased risk of developing mental disorders which are significantly associated with negative clinical outcomes and propagation of new HIV infections. There is little evidence that links inflammation to development of mental disorders among HIV patients. Therefore, the main objective of this study was to evaluate if mental health symptoms were associated with biomarkers of inflammation in HIV infected subjects.

**Methods:**

A cross-sectional study was conducted in Dar es Salam, Tanzania from March to May 2018. Standardized tools were used to collect data based on the World Health Organisation's (WHO) stepwise approach for non-communicable diseases (NCD) surveillance. A total of 407 HIV+ patients on antiretroviral therapy were recruited. The WHO stepwise approach for NCD surveillance was used to collect data together with anthropometric measurements. Mental health symptoms were determined based on self-reported thoughts of helplessness, suicide ideation, depression, despair, discouragement, and feelings of isolation. Enzyme-linked immunosorbent assay was used to test for inflammatory markers:- C-reactive protein (CRP), Iinterleukin-6 (IL-6), interleukin-18 (IL-18), soluble tumour necrosis factor receptor-I (sTNFR-I), and soluble tumour necrosis factor receptor-II (sTNFR-II). Bivariate and multi-variate analysis was conducted to examine the association between biomarkers and mental health symptoms.

**Results:**

The prevalence of self-reported mental health symptoms was 42% (*n* = 169). Participants with self-reported symptoms of mental health had elevated CRP, were less likely to walk or use a bicycle for at least 10 minutes, were less likely to participate in moderate-intensity sports or fitness activities, and had poor adherence to HIV treatment (*p* < 0.005). CRP remained significant in the sex adjusted, age-sex adjusted, and age-sex-moderate exercise adjusted models. In the fully adjusted logistic regression model, self-reported mental health symptoms were significantly associated with a higher quartile of elevated CRP (OR 4.4; 95% CI 1.3–5.9) and sTNFR-II (OR 2.6; 95% CI 1.4–6.6) and the third quartile of IL-18 (OR 5.1;95% CI 1.5–17.5) as compared with those reporting no mental health symptoms. The significance of sTNFR-II and IL-18 in the fully adjusted model is confounded by viral load suppression rates at the sixth month.

**Conclusion:**

High CRP and sTNFR II were important contributors to the prevalence of mental health symptoms. This study is among the minimal studies that have examined mental health issues in HIV, and therefore, the findings may offer significant knowledge despite the potential reverse causality. Regardless of the nature of these associations, efforts should be directed toward screening, referral, and follow-up of HIV patients who are at-risk for mental health disorders.

## Background

Human immunodeficiency virus (HIV) and mental disorders are predicted to be the leading causes of illness worldwide by the year 2030 [1]. Tremendous progress has been made globally since HIV – type 1 (HIV-1) was discovered in 1983 [2], with acquired immunodeficiency syndrome (AIDS) related annual deaths declining by 33% from 2010 to about 770,000 in 2018 [3, 4]. People living with HIV (PLHIV) face many negative experiences including stigma, discrimination, traumatic events, and mental health problems [5, 6]. These negative experiences increase the risk of developing mental disorders in PLHIV [5, 6]. HIV un-infected people with mental health problems have a higher risk of HIV infection than the general population [7–10] with severe mental illness increasing the risk of HIV acquisition by 4 to 10 fold [11]. Psychiatric disorders among HIV-infected individuals are significantly associated with negative clinical outcomes including reduced highly active antiretroviral therapy (HAART) utilization, poor adherence to HAART, and poor virological suppression [6, 12, 13]. On the other hand, mental health problems increase risky sexual behaviors like unprotected sex and multiple sexual partners which exacerbate the probability of spreading HIV [5, 6, 14]. Depressive symptoms are the most common form of mental disorders in HIV-1 infected patients and can also be a side effect of HAART [6, 13].

It is widely recognized that increased levels of inflammation are central to the development of mental health problems like depression [15–19]. Recent studies indicate a bi-directional relationship [20]. Notwithstanding, the evidence linking inflammation to mental disorders comes from observations of elevated levels of inflammatory markers in patients with mental health problems, co-occurrence of mental health problems with inflammatory illnesses, and increased risk of mental health problems with cytokine treatment [21, 22]. Following HIV-1 infection, the establishment of immune activation and inflammation involve several mechanisms that are directly or indirectly related to viral replication [23]. High levels of pro-inflammatory cytokines, such as tumour necrosis factor alpha (TNFα), interleukin 6 (IL-6) and interleukin 1 beta (IL-1β) in both plasma and lymph nodes, are observed from the early stages of HIV-1 infection [24–26]. In vitro studies suggest that HIV gene products can induce directly the activation of lymphocytes and macrophages and the production of pro-inflammatory cytokines and chemokines [23, 24]. There is evidence to support the ‘cytokine hypothesis of depression’ which implies that pro-inflammatory cytokines, acting as neuromodulators, represent the key factor in the mediation of the behavioral, neuroendocrine, and neurochemical features of depressive disorders [27]. This hypothesis was emphasized by findings of increased plasma cytokines (IL-1 and IL-6) in the blood of depressed patients [28]. More robust research measuring direct markers of inflammation and mental health is needed among HIV-1 infected patients [15, 29].

Besides HIV/AIDS, there are other risk factors for increased risk of mental disorders. The impact of chronic stress and adverse life events on the onset of depression has been investigated repeatedly, partly influenced by studies of the somatic and endocrine consequences of stress in animals [16]. There is association of severely life-threatening events prior to onset of mental disorders such as depression, anxiety disorders, and affective disorders [30–32]. However, research has not shown the expected clear-cut difference in the presence of adverse life events provoking onset in depression [33]. The prevalence of depression is higher in females than males, and this cannot be explained by differing rates of exposure to stressful life events [16, 34]. There is also evidence that genetic factors play an important role in the etiology of affective disorders [16, 35].

As there is strong evidence of deranged inflammatory response in HIV-1 as well as in mental health disorders, we set out to assess markers related to the inflammation in HIV-1 infected subjects with and without mental health symptoms. We posit that mental health symptoms lead to increased pro-inflammatory cytokine levels (CRP, IL-6, IL-18, sTNFR I, sTNFR II) in HIV-1 infected subjects.

## Methods

### Study design, site, and population

The study adopted a cross-sectional study design. Data was collected from PLHIV receiving treatment at the Infectious Disease Clinic (IDC) between March to May 2018. The Clinic is located in Dar es Salaam, Tanzania and provides care and treatment to a high proportion of PLHIV with an estimated 100 patients per day. Adults (> 18 years) living with HIV/AIDS, receiving care at IDC, and consented to be part of the study were included. The exclusion criteria was patients who were on antihypertensives and anti-inflammatory drugs within the past 3 months [36].

### Sample size and sampling technique

This study recruited 407 HIV-positive study participants on Antiretroviral Therapy (ART). Purposive sampling was used whereby HIV patients attending appointments were screened, consented, and were recruited into the study.

### Data collection

The study entailed quantitative data collection through survey, review of medical records, anthropometry, blood pressure assessments, and biochemical assessment of biomarkers in blood samples. The survey utilised the WHO Steps tool for NCD surveillance [37] to collect demographic and behavioural characteristics of participants [36]. Data on CD4 count, types, and duration of a ART was extracted from medical records. A trained nurse took blood pressure measurements as well as measurements of weight, height, and waist circumference.

### Blood pressure measurements

Diastolic and systolic blood pressure measurements were taken using an *Omron M2 (*HEM-7121-E). This is a clinically validated automated blood pressure monitor. Two measurements were taken twice from the upper left arm at 3 minute intervals when the participant was seated and relaxed [36]. An average of the two readings was calculated and used in analysis. In order to diagnose hypertension, an average reading of greater than 140/90 mmHg was used.

### Anthropometric measurements

Anthropometric measurements included waist and hip circumeference (cm), body weight (kg), and height (meters). Standard procedures of taking measurements were observed. For waist and height circumference measurements, waist to height ratio (WHR) was calculated in which > 50% was considered to represent an elevated WHR. Additionally, body mass index (BMI) was calculated based on weight and height, and the following cut-offs were used: under-weight (BMI < 18 kg/m^2)^, normal (BMI ≥ 18 kg/m^2^ and ≤ 25 kg/m^2^), overweight (BMI > 25 kg/m^2^ and < 30 kg/m^2^), and obese (BMI ≥ 30 kg/m^2^) [36].

### Blood sample collection and handling

This part of the process entailed collection of venous blood by trained phlebotomists. The blood samples were collected into Ethylenediaminetetraacetic acid (EDTA) tubes. In order to obtain blood serum, the sample collected centrifuges for 20 minutes at 1000×g, and serum obtained was stored in cryo vials at − 80 °C. Using the Enzyme Linked Immunosorbent Assay (ELISA) reader and the *Multiskan Ascent* reader 354–90,593 [36], anti-inflammatory markers, including interleukin 6 (IL-6), C-reactive protein (CRP), interleukin 18 (IL18), soluble tumour necrosis factor receptor type I (sTNFR-I) and type II (sTNFR-II), were tested. The laboratory procedures for analyzing these inflammatory markers are fully described in our related manuscript [36].

### Self-reported mental health symptoms

The outcome variable was defined as feelings of depression, suicide ideation, low energy, helplessness, despair, and isolation in the last 30 days. We used the HIV Medical Outcomes Survey (MOS-HIV) which is a widely used and accepted measure for Health Related Quality of Life (HRQOL) in the HIV population [38]. The instrument has 35 items that assess the ten dimensions of health in individuals living with HIV (mental health, quality of life, health distress, cognitive function, and energy/fatigue). The outcome variable score was collapsed and categorized as a dichotomous variable with a “Yes” if the respondent answered yes to any of the questions on mental health and “No” if they answered no to all the questions. Some of the questions asked whether the participant was feeling down, depressed or hopeless; had poor appetite or overeating; had trouble concentrating on things such as reading; and had little interest or pleasure in doing things. The MOS-HIV tool was integrated with the WHO Stepwise approach tool [39], the primary tool for this study.

### Data analysis

Microsoft Excel was used for entry of data from case reports forms and the ELISA reader. After which the data was clean and exported to STATA 14 (StataCorp, College Station, Texas) for coding and statistical analysis. Descriptive analysis of patient characteristics was performed using frequencies, percentages, median, and interquartile range. Analysis of inflammatory markers entailed stratification of the data into centiles then frequencies and percentages derived. Chi-square tests were used to determine whether there were any differences by sex and mental health status. At the multivariate level, four models, including sex-adjusted, age-sex adjusted, age-sex-moderate exercise adjusted, and the full multivariable model that included only covariates that had a priori *p*-value < 0.1 with mental health in the univariate models, were utilised. Unadjusted and adjusted odds ratios (OR) were utilised to report associations between the variables. A *p*-value < 0.05 was statistically significant.

## Result

### Participants characteristics – males vs females

We collected data from 407 participants, 99 males and 308 females, and summarized their characteristics in Table 1. Compared to females, males were less likely to be both overweight (21.4% vs 27.7%; *P* < 0.001) and obese (5.1% vs 18.6%; *P* < 0.001), respectively. There was a higher proportion of males compared to females in participants who were over 55 years (67.7% vs 59.7%; *P* < 0.037). Male participants had less prevalent abnormal waist hip ratio compared to female participants (16.3% vs 97.1%; *P* < 0.001). Compared to females, male participants had a greater prevalence of currently smoking tobacco products (9.1% vs 1%; *P* < 0.001), consumption of alcohol within past 12 months (35.5% vs 15.7%; *P* < 0.01), and consumption of alcohol within the past 30 days (19.4% vs 9.8%; *P* < 0.05).

**Table 1:** Characteristics of mental health symptoms among HIV+ patients in Tanzania

Characteristic	Male % (*n*)	Female % (*n*)	***p*** value	Has Mental Health Symptoms	No Mental Health Symptoms	***p*** value
**Total**	24.3 (99)	75.7 (308)		42.0 (169)	58.0 (233)	**0.037**
**Age Categories**						0.085
18–24 Yrs	4.0 (4)	1.0 (3)		1.2 (2)	2.1 (5)	
25–34 Yrs	7.1 (7)	6.2 (19)		3.0 (5)	9.0 (21)	
35–44 Yrs	21.2 (21)	33.1 (102)		31.4 (53)	29.6 (69)	
55 + Yrs	67.7 (67)	59.7 (184)		64.5 (109)	59.2 (138)	
**Age**			0.180			0.870
Median (IQR)	48.0. (43–54)	46.0 (42–52)		47.0 (42–53)	46.0 (41–52)	
**BMI Categories** (*n* = 405)			**< 0.001**			0.629
Underweight	4.1 (4)	8.5 (26)		8.3 (14)	6.5 (15)	
Normal	69.4 (68)	45.3 (139)		53.0 (89)	50.0 (116)	
Overweight	21.4 (21)	27.7 (85)		25.6 (43)	26.3 (61)	
Obesity	5.1 (5)	18.6 (57)		13.1 (22)	17.2 (40)	
**Waist to Hip ratio indicator**			**< 0.001**			0.099
Abnormal	16.3 (16)	97.1 (298)		81.5 (137)	74.6 (173)	
Normal	83.7 (82)	2.9 (9)		18.5 (31)	25.4 (59)	
**Years spent at school**			**< 0.001**			0.200
Median (IQR)	7.0 (7–11)	7.0 (7–7)		7.0 (7–7)	7.0 (7–9)	
**Ever consumed any alcohol** (*n* = 405)			0.286			0.264
Yes	62.6 (62)	56.5 (173)		54.8 (92)	60.3 (140)	
No	37.4 (37)	43.5 (133)		45.2 (76)	39.7 (92)	
**Consumed alcohol within 12 months?** (*n* = 234)			0.001			0.340
Yes	35.5 (22)	15.7 (27)		23.9 (22)	18.7 (26)	
No	64.5 (40)	84.3 (145)		76.1 (70)	81.3 (113)	
**Alcohol frequency within 12 months **(*n* = 49)			0.820			0.525
Daily	4.5 (1)	0 (0)		0.0 (0)	3.8 (1)	
5–6 days per week	4.5 (1)	3.7 (1)		9.1 (2)	0.0. (0)	
3–4 days per week	4.5 (1)	7.4 (2)		4.5 (1)	7.7 (2)	
1–2 days per week	27.3 (6)	29.6 (8)		22.7 (5)	34.6 (9)	
1–3 days per month	27.3 (6)	37 (10)		36.4 (8)	30.8 (8)	
Less than once a month	31.8 (7)	22.2 (6)		27.3 (6)	23.1 (6)	
**Consumed alcohol within 30 days?** (*n* = 235)			0.050			0.543
Yes	19.4 (12)	9.8 (17)		14.1 (13)	11.4 (16)	
No	80.6 (50)	90.2 (156)		85.9 (79)	88.6 (124)	

Compared to males, females were less likely to be involved in vigorous-intensity work (13.1% vs 1.6%; *P* < 0.001). Male participants were more likely to walk or use a bicycle for at least 10 minutes compared to female participants (57.6% vs 41.2%; *P* < 0.05). Compared to males, females were less likely to participate in moderate-intensity sports or fitness activities (13.1% vs 2.9%: *P* < 0.001). Males were more likely to have elevated TNR-2 as compared to females (23.3% vs 21.3%; *P* < 0.05) as indicated in Table 2.

**Table 2:** Characteristics of mental health symptoms and physical activity among HIV+ patients in Tanzania

Characteristic	Male % (*n*)	Female % (*n*)	***p*** value	Has Mental Health Symptoms	No Mental Health Symptoms	***p*** value
**Does your work involve vigorous-intensity activity?**			**< 0.001**			0.565
Yes	13.1 (13)	1.6 (5)		3.6 (6)	4.7 (11)	
No	86.9 (86)	98.4 (303)		96.4 (163)	95.3 (222)	
**Does your work involve moderate-intensity activity?**			0.280			0.564
Yes	39.4 (39)	33.4 (103)		36.7 (62)	33.9 (79)	
No	60.6 (60)	66.6 (205)		63.3 (107)	66.1 (154)	
**In a typical week, on how many days do you do moderate-intensity activities?**			0.090			**0.060**
median (IQR)	1.5 (0–6)	0.0 (0–6)		0.0 (0–6)	0.0 (0–5)	
**Do you walk or use a bicycle (pedal cycle) for at least 10 minutes continuously?**			**0.004**			**0.002**
Yes	57.6 (57)	41.2 (127)		36.7 (62)	51.9 (121)	
No	42.4 (42)	58.8 (181)		63.3 (107)	48.1 (112)	
**Do you do any vigorous-intensity sports, fitness, or recreational (leisure) activities?**			**< 0.001**			**0.019**
Yes	13.1 (13)	2.9 (9)		8.3 (14)	3 (7)	
No	86.9 (86)	97.1 (299)		91.7 (155)	97 (226)	
**Do you do any moderate-intensity sports, fitness, or recreational (leisure) activities?** (*n* = 406)			0.712			**0.014**
Yes	5.1 (5)	4.2 (13)		7.1 (12)	2.1 (5)	
No	94.9 (93)	95.8 (295)		92.9 (156)	97.9 (228)	

### Participants' characteristics – self reported mental health symptoms vs no mental health symptoms

169 (42%) participants had self-reported mental health symptoms compared to 233 (58%) participants who reported none. Participants with self-reported symptoms of mental health had elevated CRP (32.1% vs 19.1%; *P* < 0.05) compared to those without. Participants with self-reported mental health symptoms were less likely to walk or use a bicycle for at least 10 minutes compared to those without (36.7% vs 51.9%; *P* < 0.05). Compared to those with self-reported mental health symptoms, participants without mental illness were less likely to participate in moderate-intensity sports or fitness activities (7.1% vs 2.1%: *P* < 0.05) and vigorous activities (8.3% vs 3%: *P* < 0.05).

Participants with self-reported mental health symptoms had lower ART adherence in the past week (14.9% vs 4.3%; *P* < 0.05) and in the past month (18.9% vs 11.2%; *P* < 0.05) compared with those without mental health symptoms (Table 3).

**Table 3:** Characteristics of mental health symptoms and clinical variables among HIV+ patients in Tanzania

Characteristic	Male % (*n*)	Female % (*n*)	***p*** value	Has Mental Health Symptoms	No Mental Health Symptoms	***p*** value
**Baseline Viral Load** (*n* = 385)			0.826			0.333
Unsuppressed	8.3 (8)	7.6 (22)		6.3 (10)	9 (20)	
Suppressed	91.7 (88)	92.4 (266)		93.7 (148)	91 (201)	
**Current Viral Load** (*n* = 385)			0.826			0.333
Unsuppressed	8.3 (8)	7.6 (22)		6.3 (10)	9 (20)	
Suppressed	91.7 (88)	92.4 (266)		93.7 (148)	91 (201)	
**Baseline CD4** (*n* = 356)			0.56			0.376
< =350	71.9 (64)	65.8 (175)		65.4 (100)	69.2 (137)	
351–500	13.5 (12)	15.8 (42)		14.4 (22)	16.2 (32)	
> 500	14.6 (13)	18.4 (49)		20.3 (31)	14.6 (29)	
**Current CD4** (*n* = 355)			0.795			0.786
< =350	29.2 (26)	26.0 (69)		27 (41)	27.3 (54)	
351–500	28.1 (25)	27.5 (73)		26.3 (40)	29.3 (58)	
> 500	42.7 (38)	46.4 (123)		46.7 (71)	43.4 (86)	
**ART adherence in the past week** (*n* = 406)			0.146			**< 0.001**
Adhered	94.9 (94)	90.2 (277)		85.1 (143)	95.7 (223)	
Non-dherence	5.1 (5)	9.8 (30)		14.9 (25)	4.3 (10)	
**ART adherence in the past month**			0.831			**0.029**
Adhered	84.8 (84)	85.7 (264)		81.1 (137)	88.8 (207)	
**Non Adherence**	15.2 (15)	14.3 (44)		18.9 (32)	11.2 (26)	
ART Duration (Years)			0.19			0.16
Median (IQR)	6.5 (4.60–9.50)	7.45 (4.30–9.89)		7.6 (4.40–9.89)	7 (4.40–9.85)	
**ART Duration** (*n* = 406)			0.993			0.184
< 1 year	3.0 (3)	3.3 (10)		2.4 (4)	3.9 (9)	
1–3 Year	13.1 (13)	13.1 (40)		16.1 (27)	10.3 (24)	
> 3 Years	83.8 (83)	83.7 (256)		81.5 (137)	85.8 (199)	
**Type of ART** (*n* = 405)			0.998			0.060
AZT Regimen	34.3 (34)	35.0 (107)		29.8 (50)	38.8 (90)	
TDF Regimen	37.4 (37)	36.3 (111)		34.5 (58)	37.5 (87)	
D4T Regimen	22.2 (22)	22.6 (69)		28.0 (47)	18.5 (43)	
Other Regimen	6.1 (6)	6.2 (19)		7.7 (13)	5.2 (12)	

There was a significant difference in elevated CRP (*p* = 0.013) and sTNFR-II (*p* = 0.036) between participants who had mental health symptoms and no health symptoms (Table 4).

**Table 4:** Characteristics of mental health symptoms and inflammatory markers among HIV+ patients in Tanzania

Characteristic	Male % (*n*)	Female % (*n*)	***p*** value	Has Mental Health Symptoms	No Mental Health Symptoms	***p*** value
**CRP** (*n* = 404)			0.919			**0.013**
Q1	27.8 (25)	26.0 (78)		23 (38)	29.1 (64)	
Average	46.7 (42)	49.0 (147)		44.8 (74)	51.8 (114)	
Q4	25.6 (23)	25.0 (75)		32.1 (53)	19.1 (42)	
**IL 6** (*n* = 404)			0.452			0.809
Q1	24.4 (22)	28.3 (85)		27.9 (46)	26.4 (58)	
Average	52.2 (47)	44.7 (134)		44.8 (74)	48.2 (106)	
Q4	23.3 (21)	27.0 (81)		27.3 (45)	25.5 (56)	
**IL 18** (*n* = 403)			0.509			0.718
Q1	21.1 (19)	26.4 (79)		23.6 (39)	26.5 (58)	
Average	54.4 (49)	48.2 (144)		51.5 (85)	47.5 (104)	
Q4	24.4 (22)	25.4 (76)		24.8 (41)	26 (57)	
**TNR 1** (*n* = 404)			0.181			0.509
Q1	26.7 (24)	25.3 (76)		23 (38)	27.7 (61)	
Average	41.1 (37)	51 (153)		49.1 (81)	48.2 (106)	
Q4	32.2 (29)	23.7 (71)		27.9 (46)	24.1 (53)	
**TNR 2** (*n* = 404)			**0.01**			**0.036**
Q1	13.3 (12)	29 (87)		27.3 (45)	24.1 (53)	
Average	63.3 (57)	49.7 (149)		55.8 (92)	50.5 (111)	
Q4	23.3 (21)	21.3 (64)		17 (28)	25.5 (56)	

### Odds ratio tables

In the sex-adjusted model, CRP was significant (OR 2.27; 95% CI 1.24–4.16) and was also observed to be significant in the age-sex adjusted model (OR 2.14; 95% CI 1.15–3.96) and the age-sex-moderate exercise model (OR 2.14; 95% CI 1.14–4). No other significant associations between biomarkers (pro-inflammatory markers) and self-reported mental health symptoms were present after multivariable adjustment. In the full adjusted logistic regression model, those in the highest quartile (Q4) of CRP (OR 4.37; 95% CI 1.28–5.88) and TNR-2 (OR 2.61; 95% CI 1.39–6.66) had a higher odds of having mental health symptoms (Table 5).

**Table 5:** Logistic regression on mental health symptoms

Biomarker	Sex-Adjusted	***p*** value	Age-Sex Adjusted	***p*** value	Age-Sex-Moderate Exercise Adjusted Model	***p*** value	Full Model*	***p*** value
**CRP**								
Q3	1.10 (0.66–1.82)	0.717	1.08 (0.64–1.81)	0.783	1.12 (0.66–1.89)	0.673	1.11 (0.38–3.27)	0.85
Q4	2.27 (1.24–4.16)	**0.008**	2.14 (1.15–3.96)	**0.016**	2.14 (1.14–4)	**0.018**	4.37 (1.28–5.88)	**0.018**
**TNR 1**								
Q3	1.09 (0.65–1.83)	0.734	1.10 (0.65–1.86)	0.715	1.04 (0.61–1.76)	0.897	1.71 (0.53–5.5)	0.366
Q4	1.54 (0.85–2.77)	0.152	1.51 (0.84–2.73)	0.172	1.45 (0.8–2.64)	0.221	1.87 (0.55–6.36)	0.316
**TNR 2**								
Q3	1.01 (0.61–1.7)	0.958	1.06 (0.63–1.78)	0.835	1.08 (0.64–1.82)	0.783	1.48 (0.49–4.53)	0.487
Q4	1.66 (0.35–1.85)	0.202	1.71 (0.37–1.85)	0.295	1.79 (0.36–1.93)	0.266	2.61 (1.39–6.66)	**0.013**
**IL 6**								
Q3	0.84 (0.51–1.38)	0.487	0.86 (0.52–1.43)	0.566	0.90 (0.54–1.51)	0.694	1.33 (0.45–3.9)	0.609
Q4	0.81 (0.45–1.45)	0.473	0.83 (0.46–1.5)	0.544	0.84 (0.46–1.53)	0.577	0.71 (0.22–2.35)	0.577
**IL 18**								
Q3	1.32 (0.79–2.2)	0.293	1.26 (0.75–2.12)	0.378	1.24 (0.73–2.09)	0.424	5.14 (1.51–17.52)	**0.009**
Q4	1.36 (0.74–2.49)	0.316	1.26 (0.68–2.33)	0.46	1.22 (0.66–2.28)	0.522	3.30 (0.7–15.49)	0.13

*Dull model was adjusted for sex, age, moderate exercise, parent with type II diabetes, WHR, viral load copies at 6 months, vigorous-intensity sports, and weekly walking and cycling

*The reference group for all models was Q1

## Discussion

CRP has long been regarded as an acute phase protein indicative of ongoing infectious or inflammatory disease states, and is an identified marker associated with HIV disease [36, 40–44]. In a purposive sample of HIV positive Tanzanian adults, we found that elevated levels of CRP were positively associated with self-reported mental health symptoms. The observed association was independent of potential confounders such as age, WHR, physical activity, family history of diabetes, viral load (VL), and TNR2. To our knowledge, this study is the first to report on the positive association between CRP and self-reported mental health symptoms among HIV patients in Tanzania. This is consistent with data from the Korean National Health and Nutrition Examination Survey (KNH&NES) which correlated elevation in hs-CRP with mobility problems and suicidal ideation leading to decreased quality of life and mental health problems [45]. Despite other inflammatory markers (IL-6, IL-18, and sTNFR-I) assessed in our study not showing any significant association with mental health symptoms, other studies have shown a link between these markers and mental health symptoms [46–52]. Notwithstanding, none of the studies were conducted among HIV populations.

Despite the complicated and unexplored link between mental health and inflammation, activation of the sTNFR-II in patients with chronic inflammatory conditions appears to mediate depression through stimulation of a neuronal serotonin pathway leading to depletion of tryptophan [20, 53]. This may help explain the significant association between sTNFR-II levels and mental health symptoms in our study population. If uninterrupted, sustained elevations of sTNFR-II have been postulated to lead to smaller hippocampal volumes and ultimately cognitive impairment [54].

While the pathogenesis of mental illness is not clearly understood, the association of CRP, sTNFR-II, and mental health symptoms in the study population suggests an association between a proinflammatory state and psychological distress in the population [55]. This may stem from an increase in the production of kynurenic and quinolinic acids associated with activation of the enzyme indoleamine-2,3-dioxygenase that leads to a reduction in the production of serotonin, which contributes to the development of depression [56, 57]. Further, increased neurotransmitter glutamate is associated with an increase in the production of kynurenic and quinolinic acids and may result in reduced brain-derived neurotrophic factor that is widely associated with depression [58].

It is also possible that the observed association could be due to reverse causality. Having symptoms of mental health may lead to increased inflammation [59–62]. Stress hormones released during psychological stress may trigger systemic inflammation resulting in elevated levels of CRP and sTNFR-II [63]. On the other hand, HIV disease itself may serve as a trigger for inflammation that may affect mental health. Even in the era of widespread use of ART, neuropsychiatric conditions, such as anxiety disorders, manic symptoms, atypical psychosis, or depression, continue to occur in HIV patients [64, 65]. Compared to the general population, significantly higher rates of mental health disorders have been reported among PLWH elsewhere. Among PLWH in Uganda, the prevalence of major depression was 14% [66]. Approximately, 26–38% of PLWH in South Africa have a mental disorder [67]. In India and China, the prevalence of depression symptoms among PLWH were 59% and 61% of PLWH, respectively [68, 69].

Regardless of the nature of the relationship between inflammation and mental health, our study findings support the need to screen HIV infected patients for symptoms and provide follow-up care as part of of HIV care. There is a plethora of well-established and validated instruments for mental health screening that can be utilized in low-and middle-income countries (LMICs) [70]. The results also support the need for additional intervention studies aimed at improving mental health outcomes in HIV patients.

Our study was limited by lack of a validated instrument or diagnostic scoring scale to measure specific mental health outcomes such as anxiety, psychological distress, or depression. Given the cross-sectional nature of the study, we cannot establish the direction of the relationship between CRP and sTNFR-II and mental health outcomes. The main outcome of interest, symptoms of mental health, was based on self-report, which is susceptible to social desirability and self-report bias. Additionally, purposive sampling may have introduced selection bias. As such, results may not be generalizable to the entire PLWH population in Tanzania. Finally, the observed association might also be due to unmeasured confounding, such as antidepressant use, and other factors associated with mental health outcomes that was not collected for this study. Despite these limitations our study is strengthened by being amongst the pioneer studies that show an association between inflammation and mental health symptoms among PLWH from a HIV high-burden country.

## Conclusion

Our study found that in a sample of Tanzanian adults, elevated levels of pro-inflammatory cytokines, including CRP and sTNFR-II, was positively associated with self-reported mental health symptoms, independent of confounding. However, due to potential reverse causality and confounding, findings from the current study should be interpreted with caution. Regardless of the nature of these associations, efforts should be made to screen for mental health disorders in HIV patients with structured follow-up for those found at risk.

## Data Availability

Data used in the analyses for this study are available upon request from the corresponding author.

## Abbreviations

AIDS: Acquired Immunodeficiency Syndrome
ART: Antiretroviral Therapy
BMI: Body Mass Index
CI: Confidence Interval
CRP: C-reactive Protein
CRF: Case Report Forms
EDTA: Ethylenediaminetetraacetic Acid
ELISA: Enzyme-Linked Immunosorbent Assay
HAART: Highly Active Antiretroviral Therapy
HIV: Human Immunodeficiency Virus
HIV-1: Human Immunodeficiency Virus Type 1
HRQOL: Health-Related Quality of Life
IDC: Infectious Disease Clinic
IL-6: Interleukin-6
IL-18: Interleukin-18
MBL: Medical and Biological Laboratories
MOS-HIV: Medical Outcomes Survey for HIV
NCD: Non-Communicable Disease
OR: Odds Ratio
PLHIV: People Living with HIV
sTNFR I: Soluble Tumour Necrosis Factor Receptor-I
sTNFRII: Soluble Tumour Necrosis Factor Receptor-II
WHO: World Health Organization
WHR: Waist to Height Ratio
