# Correction to: Mental health symptoms and inflammatory markers among HIV infected patients in Tanzania

**DOI:** 10.1186/s12889-021-11487-0

**Published:** 2021-08-11

**Authors:** Peter Memiah, Lillian Nkinda, Mtebe Majigo, Felix Humwa, Zelalem T. Haile, Kennedy Muthoka, Aisha Zuheri, Anne Kamau, Lucy Ochola, Gabriel Buluku

**Affiliations:** 1 Division of Epidemiology and Prevention, Institute of Human Virology, University of Maryland School of Medicine, 725 West Lombard Street, Room N459, Baltimore, MD 21201 USA; 2 Department of Medicine, University of Maryland Medical Centre Midtown Campus, Baltimore, MD USA; 3 Department of Microbiology and Immunology, Muhimbili University of Health and Allied Sciences, Dar es Salaam, Tanzania; 4 Global Program for Research Teaching, University of California San Francisco, Nairobi, Kenya; 5 Department of Social Medicine, Ohio University Heritage College of Osteopathic Medicine, Dublin, OH USA; 6 Palladium Group, Nairobi, Kenya; 7 Infectious Disease Centre, Dar es Salaam, Tanzania; 8 University of Nairobi, Institute for Development Studies, Nairobi, Kenya; 9 Institute of Primate Research, Nairobi, Kenya

**Correction to: BMC Public Health 21, 1113 (2021)**

**https://doi.org/10.1186/s12889-021-11064-5**

It was highlighted that in the original article [1] the name of Zelalem T. Haile was incorrectly captured as Zelalaem Haile. The original article has been updated.
